# How to safeguard the continuous renal replacement therapy circuit: a narrative review

**DOI:** 10.3389/fmed.2024.1442065

**Published:** 2024-08-21

**Authors:** Chaomin Hu, Pengfei Shui, Bo Zhang, Xin Xu, Zhengquan Wang, Bin Wang, Jie Yang, Yang Xiang, Jun Zhang, Hongying Ni, Yucai Hong, Zhongheng Zhang

**Affiliations:** ^1^Department of Emergency Medicine, Affiliated Huzhou Hospital, Zhejiang University School of Medicine, Huzhou, China; ^2^Department of Emergency Medicine, Sir Run Run Shaw Hospital, Zhejiang University School of Medicine, Hangzhou, China; ^3^Department of Emergency Medicine, Yuyao City People's Hospital, Yuyao, China; ^4^Department of Emergency Medicine, Anji People’s Hospital, Anji, China; ^5^Department of Critical Care Medicine, Affiliated Jinhua Hospital, Zhejiang University School of Medicine, Jinhua, China; ^6^School of Medicine, Shaoxing University, Shaoxing, Zhejiang, China

**Keywords:** continuous renal replacement therapy, acute kidney injury, filter lifespan, pharmacological interventions, non-pharmacological factors

## Abstract

The high prevalence of acute kidney injury (AKI) in ICU patients emphasizes the need to understand factors influencing continuous renal replacement therapy (CRRT) circuit lifespan for optimal outcomes. This review examines key pharmacological interventions—citrate (especially in regional citrate anticoagulation), unfractionated heparin (UFH), low molecular weight heparin (LMWH), and nafamostat mesylate (NM)—and their effects on filter longevity. Citrate shows efficacy with lower bleeding risks, while UFH remains cost-effective, particularly in COVID-19 cases. LMWH is effective but associated with higher bleeding risks. NM is promising for high-bleeding risk scenarios. The review advocates for non-tunneled, non-cuffed temporary catheters, especially bedside-inserted ones, and discusses the advantages of surface-modified dual-lumen catheters. Material composition, such as polysulfone membranes, impacts filter lifespan. The choice of treatment modality, such as Continuous Veno-Venous Hemodialysis (CVVHD) or Continuous Veno-Venous Hemofiltration with Dialysis (CVVHDF), along with the management of effluent volume, blood flow rates, and downtime, are critical in prolonging filter longevity in CRRT. Patient-specific conditions, particularly the type of underlying disease, and the implementation of early mobilization strategies during CRRT are identified as influential factors that can extend the lifespan of CRRT filters. In conclusion, this review offers insights into factors influencing CRRT circuit longevity, supporting evidence-based practices and suggesting further multicenter studies to guide ICU clinical decisions.

## 1 Introduction

An estimated 57.3% of individuals admitted to the ICUs meet the criteria for acute kidney injury (AKI) (1). Continuous renal replacement therapy (CRRT) is preferred over intermittent renal replacement therapy (IRRT) in ICUs for its gentle impact on hemodynamics and solute clearance, running continuously over 24 h to optimize treatment without disrupting patient stability (2). Maximizing CRRT filter lifespan is crucial (3) as it directly influences treatment efficacy and reduces costs associated with frequent circuit changes. A compromised filter can diminish therapeutic efficacy by impeding solute clearance and necessitate more frequent circuit changes (4), impacting treatment costs (5). There are objective indicators for monitoring the lifespan and obstruction of CRRT circuits (6–9). The key parameters include access pressure (AP), prefilter pressure (FP), transmembrane pressure (TMP), effluent pressure (EP), and return pressure (RP). Each 1-mmHg rise in TMP or filter pressure independently increases clotting risk by 1.5% (95% CI 1.0–2.0%) (6). A significantly negative AP (≤ −200 mm Hg) has been correlated with circuit failure within 12 h (7). TMP values above 300 mmHg indicate a need for potential filter replacement (9). Vigilant monitoring of these indicators allows proactive measures against circuit obstruction, thereby prolonging CRRT circuit life and ensuring therapy continuity and efficacy. While not universally adopted in current CRRT guidelines, AP, FP, TMP, EP, and RP provide valuable insights for clinicians, supporting optimized clinical practices (10–14). Future large-scale randomized controlled trial (RCT) studies are anticipated to validate these indicators for broader inclusion in clinical guidelines. Therefore, understanding and optimizing the CRRT filter lifespan is crucial for ensuring the therapy’s effectiveness and reducing unnecessary expenses.

The KDIGO guidelines state that the use and replacement of filters should be based on monitored filter performance (such as TMP and blood flow) and patient clinical condition assessment. There are no recommended specific standardized durations or removal criteria; decisions should be made based on individual circumstances, with particular attention to extracorporeal circuit clotting as a primary cause for unplanned filter replacement (10). The recurrent occurrence of clotting curtails the precious duration of therapeutic intervention, leading to suboptimal treatment outcomes and amplifying the economic burden of treatment and the workload for healthcare personnel. Upon the occurrence of coagulation in the CRRT circuit, a substantial depletion of the patient’s platelets ensues, consequently increasing the risk of mortality (15). Coagulation also substantially contributes to hemodynamic losses in patients, necessitating an increased demand for transfusions (16, 17). Therefore, enhancing filter longevity and performance efficiency in CRRT has been the subject of numerous recent researches. Methods to prolong the lifespan of the circuit encompass both pharmacological interventions and non-pharmacological factors. In terms of pharmacological interventions, larger, high-quality studies have primarily focused on determining optimal anticoagulation strategies, and this aspect has been central to several reviews (3, 5, 18). Attempts that are made in the ICU to prevent filters from clotting are not limited to pharmacological interventions. In CRRT, determining the optimal vascular access configuration involves various factors such as catheter design, size, insertion site, inserter experience, insertion depth, and line maintenance. Inappropriate access may lead to frequent alarms on the CRRT platform, resulting in treatment delivery failures or reduced blood flow, thereby affecting the effectiveness of therapy and encouraging stasis, potentially leading to thrombosis (19–22). Factors related to the patient, including underlying conditions, patient pathology, and illness severity, collectively influence the ease of conducting CRRT and preserving vascular access (23–26). Circuit-related variables encompass factors associated with the blood filtration membrane, such as polyamide, polysulfone, or polyethylene, as well as treatment modalities including commonly utilized CVVHD, continuous veno-venous hemofiltration (CVVH), and CVVHD-F in clinical settings (27). Practices in this field also involve various approaches, such as modifications in the implementation of pre/post-dilution in CVVH and CVVHD-F, effluent volume, target blood fluid flow rates, and overall circuit management procedures (28).

This review will comprehensively survey methods for prolonging the lifespan of CRRT circuits, covering both pharmacological interventions and non-pharmacological factors.

## 2 Methods

### 2.1 Scope and coverage of literature search

Our review focuses on exploring the filter lifespan in CRRT for AKI, along with the effects of pharmacological and non-pharmacological interventions. The search was limited to strategies aimed at prolonging filter lifespan in CRRT.

### 2.2 Keywords and search strategy

We utilized a comprehensive search strategy incorporating keywords such as “continuous renal replacement therapy,” “acute kidney injury,” “filter lifespan,” “pharmacological interventions,” and “non-pharmacological factors.” these keywords were combined using Boolean logic to ensure thorough coverage of relevant literature.

### 2.3 Database selection

We conducted searches across PubMed and Google scholar due to their extensive coverage of biomedical literature and multidisciplinary approach.

### 2.4 Time frame

Our search spanned articles published between January 2005 and December 2023 to capture the most relevant and recent studies.

### 2.5 Types of literature

We included peer-reviewed original studies, review articles, case studies, systematic reviews, and meta-analyses in our review.

### 2.6 Language restrictions

We limited our search to English-language publications to maintain consistency and clarity in the reviewed literature.

### 2.7 Screening process

Initially, articles were screened based on titles and abstracts to exclude irrelevant studies. Subsequently, full-text reviews were conducted to determine eligibility based on relevance to our review objectives.

### 2.8 Quality assessment

Selected studies underwent rigorous quality assessment, considering study design, sample size, result consistency, and risk of bias.

### 2.9 Updated searches

To ensure completeness, additional searches were conducted following the initial submission to incorporate the latest research findings.

### 2.10 Data management and recording

We maintained detailed records of our search results and screening processes using Zotero literature management software for systematic data organization.

## 3 Pharmacological interventions

**Figure 1:**
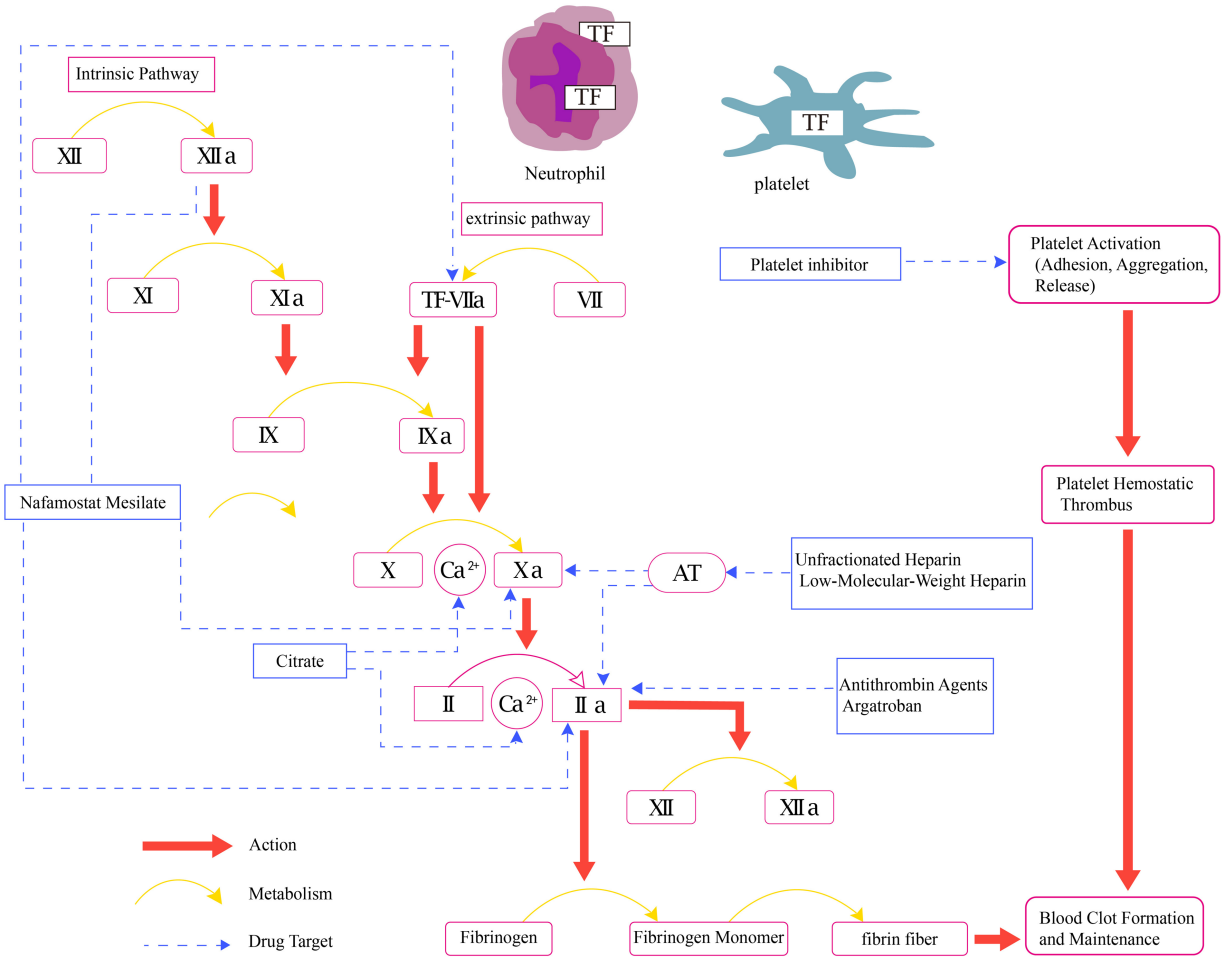
Physiology of coagulation and mechanisms of pharmacological interventions in CRRT. AT, anticoagulant blood count; Ca2+, calcium ion; TF, tissue factor; II, prothrombin; IIa, activated factor II; V, labile factor; VI, accelerin; VII, proconvertin; VIIa, activated factor VII; VIII, antihemophilic factor; IX, christmas factor; IXa, activated factor IX; X, stuart-prower factor; Xa, activated factor X; XI, plasma thromboplastin antecedent; XIa, activated factor XI; XII, Hageman factor; XIIa, activated factor XII.

Pharmacological interventions can impact various stages within the cascade of coagulation in the human body (Figure 1), thereby interrupting the formation of blood clots. Pharmacological approaches encompass the use of a range of medications, including intravenous anticoagulants (such as UHF, LMWH, argatroban, bivalirudin, and RCA), oral anticoagulants (warfarin), and antiplatelet agents.

Citrate exerts its effects through the chelation of calcium, thereby inhibiting calcium-dependent steps in the biochemical coagulation process (29). One reason we prefer RCA is its ability to maintain effective circuit anticoagulation without increasing the risk of bleeding. This advantage makes RCA a commonly used anticoagulation method in CRRT (30–32). The Kidney Disease: Improving Global Outcomes (KDIGO) guidelines recommend using RCA rather than UHF in patients who do not have contraindications for RCA (10). Several RCTs have shown a significant prolongation of filter lifespan in the RCA group (9, 16, 33). A recent RCT (34) involving 596 patients reported a significantly prolonged filter lifespan in the RCA group (unadjusted median, 46.5 h [IQR, 18.8–70.3 h] vs. 26.0 h [IQR, 12.0–50.6 h]; unadjusted absolute difference, 11.6 h [95% CI, 8.5–14.7 h]; adjusted absolute difference, 11.2 h [95% CI, 8.2–14.3 h]; *p* < 0.001). Notably, bleeding complications were significantly reduced in the RCA group compared to the UHF group (5.1% vs. 16.9%; *p* < 0.001; OR, 0.27 [95% CI, 0.15–0.49]). The systematic reviews by Tsujimoto H suggest that, in comparison to UHF, RCA may reduce the risk of thrombocytopenia (RR 0.39, 95% CI 0.14 to 1.03; low certainty of evidence) (5). This reduction could be attributed to a decrease in heparin-induced thrombocytopenia (HIT), a common complication of heparin anticoagulation (35). In CRRT treatment for high bleeding risk patients, such as those post-cardiac surgery (26, 36) or with severe trauma (37), RCA therapy demonstrates improved safety and hemodynamic stability. However, the incidence of new culture-proven infection since initiating dialysis was higher in the RCA group (68.0% vs. 55.4%; *p* = 0.002; OR, 1.71 [95% CI, 1.23 to 2.39]) (34). This increase may be related to factors such as enhanced monitoring and prolonged filter lifespan. Additionally, hypophosphatemia associated with RCA may compromise the immune response, potentially increasing the risk of infection. The observed trend of a higher infection rate in the RCA group merits in-depth investigation in subsequent trials. Diagnosing citrate accumulation remains a relatively intricate task when there is a lack of monitoring for citrate concentration. The primary objective of RCA protocols should be to minimize the net citrate load administered to patients (38). Throughout treatment, it is crucial to closely monitor ionized calcium and perform regular assessments of total/ionized calcium and pH values. Employing limited blood flow is recommended, and a preference for high dialysate/filtration rates should be considered to optimize the removal of citrate (Table 1).

**Table 1:** Characteristics of studies reporting RCA with filter life (5, 9, 16, 30, 32–34, 36, 37, 39).

Study name	Sample size	Design	Description	Inclusion criteria	Exclusion criteria	Intervention/Comparison	Control
Tsujimoto H et al. 2016 (5)	412 patients/unclear	Retrospective review	RCA vs. UHF	Up to 12 September 2019, selected randomized controlled trials (RCTs or cluster RCTs) and quasi-RCTs of pharmacological interventions to prevent clotting of extracorporeal circuits during CRRT	No	RCA	UHF
Fabien Stucker et al. 2015 (9)	103 patients/unclear	RCT	Citrate-based anticoagulation vs. heparin	ICU patients were eligible if they were ≥ 18 years of age and had an AKI requiring CRRT according to the kidney failure criteria of the RIFLE definition.	Patients were excluded if they had active hemorrhagic disorders or severe thrombocytopenia (<50 × 109/L), a history of heparin-induced thrombocytopenia, severe liver failure defined as a factor V < 20%, or were on the waiting list for liver transplantation.	RCA	Heparin
Gattas DJ et al. 2015 (16)	212 patients/857 filters	RCT	RCA vs. regional anticoagulation using heparin and protamine	(1) Acute renal failure requiring CRRT, (2) Suitability for regional anticoagulation of the CRRT circuit, (3) Clinical equipoise regarding the method of circuit anticoagulation, and (4) Informed consent was given or sought soon after enrollment.	(1) Expected stay in ICU less than 24 h, (2) Age less than 18 years, (3) Pregnant or breastfeeding, (4) Suspected ischemic hepatitis or liver failure, (5) Known allergy to heparin or protamine, (6) Suspected or confirmed heparin-induced thrombocytopenia (HIT), and (7) Chronic kidney disease requiring dialysis before ICU admission.	RCA with the maintenance of systemic normocalcemia	Regional heparin anticoagulation with protamine reversal
Bai M et al. 2023 (30)	89 patients/unclear	RCT	RCA vs. no-anticoagulation	(1) Liver failure (acute liver failure and chronic liver failure), (2) High bleeding risk, (3) Scheduled CRRT treatment, and (4) Informed consent.	(1) Use of other anticoagulants, (2) Uncorrectable hypoxemia (PaO2 < 60 mmHg) or systemic hypoperfusion shock, (3) Pregnancy or lactation, and (4) Fistula, CRRT treatment time < 12 h.	RCA	No anticoagulation
Schultheiß C et al. 2012 (32)	28 patients/43 filters	Systematic review and meta-analysis	Investigate the predictive capability of baseline liver function parameters regarding citrate accumulation	Liver failure patients in the ICU receiving CRRT	Severe alkalosis (pH > 7.55) or acidosis (pH <7.1) and deficiency of ionized calcium (Caion <0.9 mmoL/L).	Characterize predictors for citrate accumulation in terms of a Catot/Caion ratio of ≥2.5 and investigate the feasibility of citrate anticoagulation in patients with markedly impaired liver function.	Controlling for other various
Louise Schilder et al. 2014 (33)	139 patients/unclear	RCT	Citrate anticoagulation vs. systemic heparinization	Patients in the ICU receiving CVVH	The presence of an increased bleeding risk (defined as a platelet count below 40 × 109/L, an activated partial thromboplastin time (aPTT) longer than 60 s, a prothrombin time-international normalized ratio (PT-INR) greater than 2.0 or recent major bleeding), age below 18 or over 80 years, the need for therapeutic systemic anticoagulation (heparin or coumarins) or a known HIT.	RCA	Systemic heparinization
Zarbock A et al. 2020 (34)	596 patients/unclear	RCT	RCA vs. systemic heparin anticoagulation	(1) KDIGO stage 3 acute kidney injury classification (urine output <0.3 mL/kg/h for ≥24 h, and/or > 3-fold increase in serum creatinine level compared with baseline, and/or serum creatinine level of ≥4 mg/dL [353.6 μmol/L] with an acute increase of at least 0.5 mg/dL [44.2 μmol/L] within 48 h) OR an absolute indication for continuous kidney replacement therapy (serum urea levels >150 mg/dL, serum potassium levels >6 mmol/L, serum magnesium levels >9.7 mg/dL [4 mmol/L], blood pH <7.15, urine production <200 mL/12 h or anuria, or fluid overload with edema in the presence of acute kidney injury resistant to diuretic treatment); (2) at least 1 additional condition (severe sepsis or septic shock, use of vasopressor, refractory fluid overload); (3) age between 18 and 90 years; (4) intention to provide full intensive care treatment for at least 3 days; and (5) provision of written informed consent.	(1) Increased Bleeding Risk: Individuals with a heightened risk of bleeding were excluded. (2) Diseases with Hemorrhagic Diathesis: Patients with conditions or organ damage associated with a tendency to bleed excessively (hemorrhagic diathesis) were excluded. (3) Need for Therapeutic Anticoagulation: Those requiring therapeutic anticoagulation were excluded. (4) Previous Allergic Reactions to Anticoagulants: Patients who had experienced allergic reactions to anticoagulants in the past were excluded. (5) Known Heparin-Induced Thrombocytopenia: Individuals with a confirmed history of heparin-induced thrombocytopenia were excluded. (6) Persistent and Severe Lactic Acidosis: Exclusion criteria included persistent and severe lactic acidosis, defined as a pH <7.2 in two consecutive measurements for more than 2 h and a lactate level > 72.1 mg/dL (8 mmol/L). This was considered in the context of acute liver failure.	RCA	Systemic heparin anticoagulation
Agnieszka Kośka et al. 2022 (39)	52 patients/193 filters	A prospective observational study	This study assesses RCA efficacy in patients admitted to critical care following cardiovascular surgery and the influence of standard antithrombotic agents routinely used in this specific group.	The inclusion of consecutive cardiovascular surgery patients treated with post-dilution hemofiltration with RCA enhances the generalizability of the findings to this specific patient population.	UFH infusion	RCA	Standard antithrombotic agents (acetylsalicylic acid, low molecular weight heparin, fondaparinux)
Morabito S et al. 2012 (36)	33 patients/302 filters	An observational study	The study aims to evaluate the efficacy and safety of RCA-continuous veno-venous hemofiltration (CVVH) using a low-concentration	(1) Patients undergoing continuous renal replacement therapy (CRRT) due to acute kidney injury (AKI) following cardiac surgery. (2) Patients deemed to have a high risk of bleeding, possibly resulting from factors such as recent cardiac surgery. (3) Patients willing to undergo regional citrate	(1) Patients not eligible for anticoagulation choices of RCA, heparin, or no anticoagulation (no-AC). (2) Presence of clinical conditions or contraindications unsuitable for RCA or other anticoagulation treatments. (3) Failure to meet defined criteria for high bleeding risk, such as a platelet count below 50,000/μl or heparin-	RCA-CVVH using a 12 mmoL/L citrate solution.	heparin or no anticoagulation
			citrate solution in critically ill patients with severe acute kidney injury following cardiac surgery.	anticoagulation (RCA) as the anticoagulation method for CRRT. (4) Written informed consent obtained from the patient or a close relative.	induced thrombocytopenia. (4) Specific clinical conditions unsuitable for RCA, such as a baseline aPTT exceeding 45 s or recent surgery within the past 48 h. (5) Occurrence of filter clotting within 24 h.		
Mariano F et al. 2023 (37)	60 patients/ unclear	A retrospective study	RCA vs. heparin anticoagulation in severe polytrauma patients with an early AKI requiring renal replacement therapy.	(1) Adult patients (≥18 years) admitted to the emergency department of CTO Hospital, Turin, with polytrauma between January 2000 and December 2021. (2) Among these patients, those who required kidney replacement therapy (KRT) during the first 72 h after admission. (3) Patients who did not have associated burns or were not treated with early CRRT due to chronic regular dialysis.	(1) Polytrauma patients with associated burns. (2) Patients treated with early CRRT due to chronic regular dialysis. (3) Patients not meeting the inclusion criteria for KRT within the specified timeframe. (4) Patients not treated with either regional citrate anticoagulation (RCA) or unfractionated heparin for anticoagulation during CRRT.	RCA	Heparin

UFH has been the primary method of anticoagulation in CRRT for decades (40), despite lacking significant superiority over RCA in prolonging filter lifespan and preventing bleeding events (16). However, healthcare professionals have extensive experience in its usage, contributing to streamlining treatment procedures and enhancing safety. For patients with contraindications to citrate, KDIGO (10) recommends using UFH, providing rapid reversibility and economic feasibility. UFH’s anticoagulant effect can be monitored and adjusted using coagulation times such as activated partial thromboplastin time (APTT), and its reversibility allows for prompt action in urgent situations such as surgeries or bleeding management. Moreover, UFH is relatively cost-effective, making it an economically viable option for patients requiring prolonged CRRT treatment. Additionally, over the past few years, a multitude of studies have emerged, investigating the use of UHF as an anticoagulant in COVID-19 patients necessitating continuous renal replacement therapy (26, 41–45). Among these studies, Endres’ research (44) found that the filter lifespan in COVID-19 ICU patients undergoing CRRT is comparatively shorter, averaging 17 h, in contrast to the control group of non-COVID-19 pulmonary infectious shock patients, where it is 39 h. Furthermore, he demonstrated that implementing a systemic heparin administration protocol guided by anti-factor Xa levels in CRRT effectively prolongs the filter lifespan (24 [15.1, 54.2] vs. 17.3 [9.5, 35.1] h, *p* = 0.04). In summary, UHF remains the primary anticoagulant due to its rapid reversibility, cost-effectiveness, and extensive healthcare professional experience. Furthermore, it exhibits notable advantages in prolonging the CRRT filter lifespan for COVID-19 patients.

In addition to conventional heparin anticoagulation, literature has also documented the use of LMWH for anticoagulation in CRRT. The filter lifespans between LMWH and UFH groups (43+/−15 vs. 52+/−18 h, *p* = 0.10), with no significant difference in circuit clotting rates. However, LMWH poses a higher risk of major bleeding compared to UFH (5). Despite lacking distinct advantages in the general population, LMWH shows benefits for COVID-19 patients. Reports (46, 47) suggest COVID-19 patients undergoing CRRT experience higher rates of premature filter change and longer dialysis downtime. In Arnold et al. (41) comparison of different anticoagulation strategies in the treatment of 71 critically ill patients with COVID-19 undergoing CRRT, the average treatment duration was 8.1 h (SEM: ±1.3 h) with UFH, 8.0 h (SEM: ±0.9 h) with argatroban, and 11.8 h (SEM: ±0.5 h) with LMWH. Notably, LMWH exhibited a significant extension in treatment duration by 3.7 h (*p* = 0.008) and 3.8 h (*p* = 0.002), respectively. Overall, LMWH demonstrates specific benefits for COVID-19 patients, as evidenced by the significant extension in CRRT filter lifespan in this study. However, it remains unclear from current literature whether CRRT anticoagulant therapy consistently involves concurrent use of LMWH or other anticoagulants. Considering this gap, we recommend future research, particularly large-scale RCTs, to further explore the impact of different anticoagulation strategies on CRRT circuit longevity in COVID-19 patients.

NM, initially designed as a synthetic serine protease inhibitor for pancreatitis treatment, has seen increased utilization in CRRT since 1990, predominantly in Japan. It inhibits platelet aggregation and various coagulation factors, including thrombin, Xa, XIIa, kallikrein, and components of the complement system. NM has demonstrated efficacy in prolonging filter lifespan, thereby improving filtration efficiency. With its versatile application and the absence of absolute contraindications, NM emerges as a suitable anticoagulant for CRRT, particularly in patients susceptible to bleeding risks (48–50). In a 2014 RCT led by Yong Kyu Lee, the efficacy of NM and no anticoagulation was assessed in high-risk bleeding patients undergoing CRRT. The study found no statistically significant difference in mortality, transfusion, or survival between the two groups, and no adverse events related to NM were reported. However, notable distinctions emerged when comparing the NM group to the no-anticoagulation group. The NM group exhibited a significantly reduced overall number of CRRT filters used (2.71 ± 2.12 vs. 4.50 ± 3.25, *p* = 0.042) and a decreased frequency of filter changes due to clots per 24 h (1.15 ± 0.81 vs. 1.74 ± 1.62, *p* = 0.040). Subdividing filter lifespan into below and over 12 h, the NM group demonstrated a significantly higher number of filters functioning beyond 12 h (*p* = 0.037, odds ratio 1.84) (48). Another smaller-sample, single-center, prospective randomized study also observed that, compared to the control group, the NM group exhibited significantly prolonged filter lifespan and enhanced filtration efficiency (51). However, larger-scale randomized trials are needed to validate its safety and effectiveness in high-bleeding risk patients compared to other anticoagulants such as citrate.

## 4 Non-pharmacological factors

**Figure 2:**
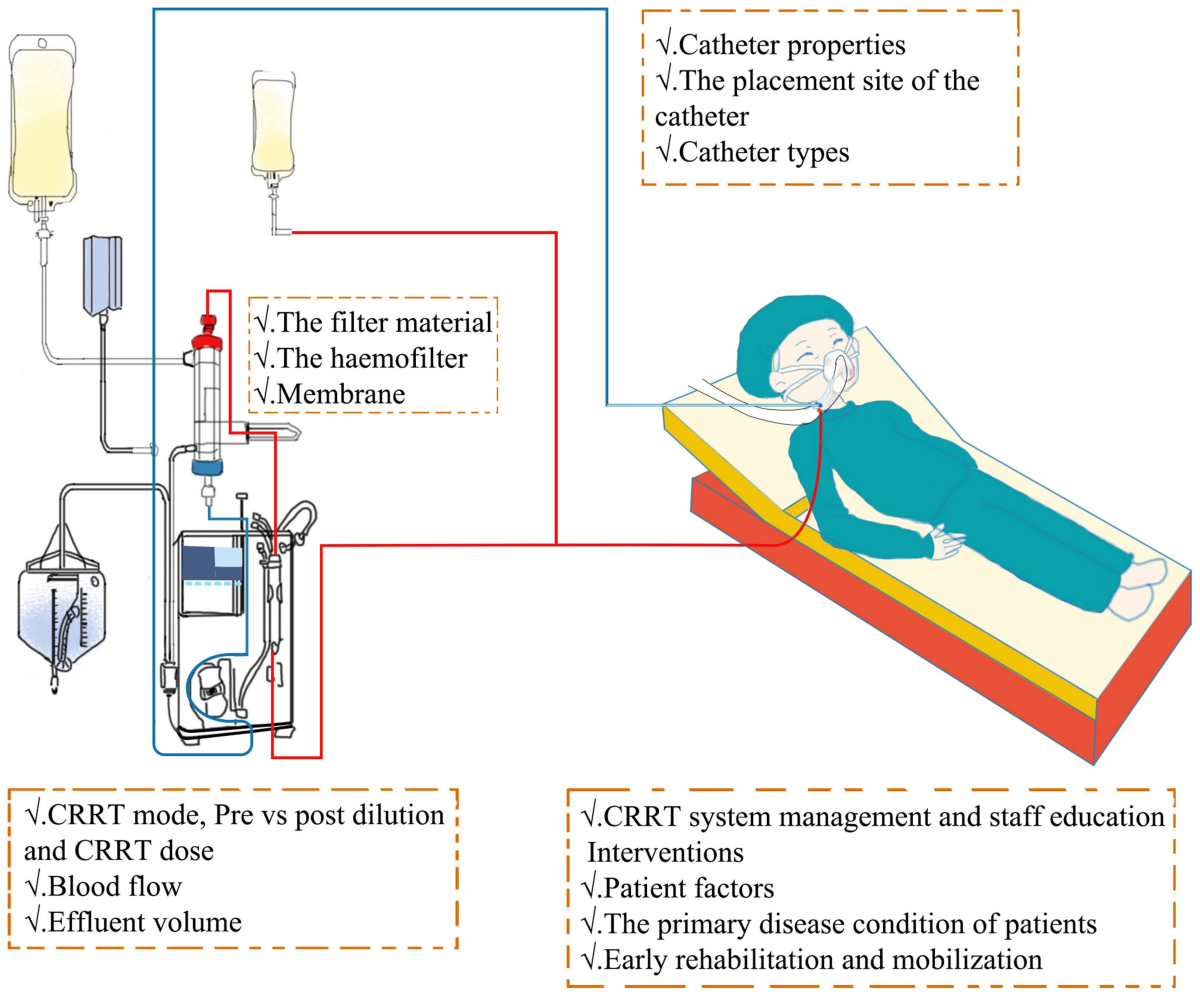
Intervention mechanism of non-pharmacological factors in CRRT. CRRT, continuous renal replacement therapy.

In addition to selecting pharmacological interventions, several non-pharmacological factors play a pivotal role in influencing the lifespan of CRRT filters (Figure 2). Coagulation in CRRT circuits may stem from variables, such as blood flow stasis or turbulence, hemoconcentration, or activation of the intrinsic coagulation system due to contact with blood air or the blood filter (52). As a result, non-pharmacological strategies aimed at preventing clotting in CRRT circuits involve meticulous catheter or entry site selection, optimization of blood flow rates, careful consideration of CRRT modalities, and implementation of blood dilution methods.

First of all, the selection of catheters significantly impacts the patency of the extracorporeal circuit in CRRT (53). For critically ill patients, using a non-tunneled, non-cuffed temporary catheter is recommended due to its ease of bedside insertion and superior survival rates compared to alternative catheters (54, 55). Klouche et al. (56) findings indicate that a non-tunneled, non-cuffed temporary catheter not only exhibited a shorter insertion time (23.2 ± 13.8 vs. 45.7 ± 27.5 min, *p* < 0.05) but also demonstrated superior catheter survival rates compared to the alternative catheter at specified time points (100% vs. 60%, *p* < 0.05). Additionally, the investigations conducted by Dunn and Sriram (57), Gilmore et al. (22), and Fealy et al. (58) explored the influence of various dialysis catheters, revealing no statistically significant association between the lifespan of the filter circuit and the type of vascular catheter utilized. In Fealy et al. (58) investigation, 26 patients (140 circuits) employed the Niagara catheter, while 20 patients (114 circuits) selected the Medcomp catheter. The circuit lifespan associated with the Niagara catheter proved markedly superior to that of the Medcomp catheter, with respective durations of 11 h and 7.3 h (*p* < 0.01). In a recent study (59), the performance of surface modified dual lumen catheters (smDLC) and standard dual lumen catheters (sDLC) was compared in 236 critically ill patients undergoing CVVHDF, indicating that smDLC exhibited superior performance in terms of prolonged duration before removal (131 ± 38 vs. 113 ± 21 h, *p* = 0.004), lower rates of temporary catheter dysfunction (5% vs. 14%, *p* = 0.001) and thrombosis (2.3 episodes per 1,000 TC-days,4.2 episodes per 1,000 TC-days, *p* = 0.021), higher blood flow rates (221 ± 29 vs. 187 ± 36 mL/min, *p* = 0.012), and a lower relative risk of premature removal [0.43 (95% CI, 0.13–0.98, *p* = 0.041)] compared to sDLC [2.51 (95% CI, 1.04–9.22, *p* = 0.034)], highlighting its potential clinical advantages over sDLC. However, caution is warranted due to the increased risk of catheter-related bacteremia associated with sDLC (*p* = 0.008). Cumulatively, the literature suggests that a nuanced approach to catheter selection is paramount for maintaining extracorporeal circuit patency and optimizing the prospects of long-term recovery.

Second, the placement site of the catheter can also influence the lifespan of the filter. Traditionally, the internal jugular vein has been preferred for vascular access in CRRT due to its association with lower catheter dysfunction and infection risks (60, 61). However, following the CATHEDIA study (62), a multicenter RCT involving patients undergoing intermittent hemodialysis or CRRT, there have been reports suggesting a comparable risk of infection, catheter dysfunction, and dialysis performance between femoral and right jugular vein catheterization. Brian’s analysis (27) indicated a shorter filter lifespan with the subclavian route compared to the femoral vein, while temporary internal jugular vein catheters showed no significant difference. Recent guidelines now recommend both femoral and right jugular veins as the initial sites for vascular access in critical care settings, while discouraging the use of left jugular and subclavian veins (62–64). In practice, the femoral vein is preferred for CRRT, particularly in sicker patients due to its faster and more successful vascular access. Dugué AE mentioned in their report that, compared to internal jugular vein catheterization, although there was no difference in the first attempt success rate on the side (63.9% vs. 62.3%, *p* = 0.88), the femoral vein catheterization required less time (10 min vs. 12 min, *p* < 0.01) (63). Additionally, catheter tip location impacts dysfunction risk, with placement in the right atrium or inferior vena cava reducing it (65, 66). Selecting an appropriately sized dialysis catheter is crucial, with catheters ideally placed in the superior vena cava (SVC) or inferior vena cava (IVC) via the recommended routes. During catheter placement, bedside ultrasound enables precise assessment and adjustment of the catheter tip position to optimize placement accuracy. Moreover, the tunneled access (14.5Fr) tended to be associated with a longer filter lifespan, and a direct connection with extracorporeal membrane oxygenation (ECMO) provided the longest filter lifespan (27, 67, 68). In summary, the choice between the femoral and right jugular veins, along with optimal catheter tip positioning, plays a crucial role in the lifespan of the filter and the overall effectiveness of CRRT. This highlights the importance of adhering to the latest guidelines in the critical care setting to enhance vascular access outcomes.

Several studies emphasize the importance of considering the characteristics of hemofilter membranes in the lifespan of CRRT circuits. These filters are typically composed of materials such as polyamide, polysulfone, or polyethylene. These materials exhibit excellent biocompatibility and dialytic performance, effectively removing waste products and excess fluids from the body during the CRRT process. One multiple regression analysis revealed a trend indicating longer filter life associated with polysulfone membranes compared to cellulose triacetate (69). Another study found no significant disparity in filter life between the newer surface-treated AN69ST membrane and a polysulfone membrane. The number of sessions interrupted for circuit clotting was 8 (15%) with AN69ST and 10 (19%) with polysulfone (*p* = 0.60) (70). Interestingly, research indicated that the AN69ST membrane, despite its heparin-binding properties, did not outperform non-surface-treated AN69 membranes in CRRT without anticoagulation regarding filter life (71). For other types of filters, including those with more fibers and shorter fibers, hollow fiber or flat fiber filters, as well as filters with larger membrane surface areas, there is no reliable evidence indicating their impact on filter life (72). Currently, investigations into filter materials predominantly rely on single-center studies with limited sample sizes, posing challenges in delivering robust evidence for evidence-based medicine. There is a pressing demand for extensive multicenter RCTs to substantiate these observations.

The choice of treatment modality plays a significant role in determining the lifespan of CRRT circuits. While clinical needs primarily dictate modality choice, it is important to note that different modalities can impact circuit longevity. Existing studies predominantly recommend CVVHDF or CVVHD over CVVH, as it significantly prolongs filter lifespan (73, 74). Recently, Mann et al. (74) reported a median filter life (with interquartile range) of 21.8 (11.4–45.3) for pre-filter CVVH, compared to 26.6 (13.0–63.5) for CVVHD, with a higher percentage of filters remaining active for over 72 h in the CVVHD group (11.8% vs. 21.2%). Currently, there is a lack of large RCTs investigating the impact of pre- and post-dilution on filter life in CVVH. The conclusions drawn from the existing three studies are still inconsistent (75–77). van der Voort et al. (75) and de Pont et al. (76) suggest that pre-dilution CVVH exhibits a longer filter run time (FRT) compared to post-dilution CVVH. However, Nurmohamed et al. (77) argues that there is no significant difference between the two. The filter life (median ± interquartile range [IQR]) in predilution modes was 24 ± 38 h, and in post-dilution modes, it was 29 ± 46 h (*p* = 0.58). In summary, the existing research on the correlation between treatment modalities in CRRT and filter lifespan is characterized by a lack of high-quality evidence. Consequently, there is an immediate imperative for extensive multicenter RCTs to furnish high-quality evidence in the realm of evidence-based medicine.

In considering the intricate interplay between treatment parameters in CRRT, the relationship among “Effluent volume,” blood flow, and filter lifespan holds significant clinical relevance. “Effluent volume” refers to the total amount of waste and solutes removed from the patient’s body during the treatment process in CRRT. In 2009, a comprehensive RCT (78) revealed that increasing CRRT effluent volume from 25 to 40 mL/kg/h did not reduce mortality or dialysis dependence. Meanwhile, they utilized 0.93 ± 0.86 filters per day in the high-intensity group, as opposed to 0.84 ± 0.81 in the lower-intensity group (*p* < 0.001). In Castillo’s study (79), he observed that there was no difference in filter lifespan between groups with an effluent volume greater than 25 mL/kg per h and those with less than 25 mL/kg per h. Prescribing a treatment intensity exceeding 25 mL of effluent flow per kg per h is unlikely to yield significant benefits. On the contrary, it may shorten filter life and even expose patients to the risk of hypophosphatemia. Additionally, existing studies suggest (57, 80) that blood flow rate influences the lifespan of the filter. In CRRT, maintaining an optimal blood flow of 200 mL/min through the hemofilter is considered ideal. Circuit clotting is a concern if blood flow falls below 100 mL/min (81) while exceeding 300 mL/min can shorten the filter circuit lifespan (57). Hence, selecting an appropriate blood flow velocity is crucial to prevent premature clotting due to filter fiber resistance.

Effective teamwork plays a pivotal role in prolonging the lifespan of vascular conduits. “Continuous” therapy is not truly continuous. Uchino et al. reported a median downtime of 3.0 h (1.0–8.3) in patients undergoing CRRT (82). Therefore, prescribing physicians need to be mindful of the impact of downtime on the quality of renal replacement therapy and filter lifespan. Simultaneously, the provision of high-quality CRRT requires the management of a highly skilled team. With the implementation of simulation-based education for CRRT training, Mottes observed a sustained, clinically, and statistically significant increase in filter life (66.4 h vs. 59.4 h, *p* = 0.008) (83). A well-organized specialized CRRT team could enhance clinical outcomes by improving the quality of care for patients requiring CRRT treatment in the ICU (84). In summary, simulation-based education in CRRT training resulted in a sustained increase in filter life, while the presence of a well-organized specialized CRRT team demonstrated potential benefits in improving clinical outcomes for ICU patients undergoing CRRT treatment.

Additionally, the primary disease condition of patients is another crucial factor influencing CRRT filter lifespan. As mentioned earlier, the lifespan of CRRT filters is notably reduced in COVID-19 ICU patients compared to a control group of non-COVID-19 patients with pulmonary infectious shock (44). In addition, Agarwal et al. (85) conducted a study comparing filter lifespan among patients with acute liver failure, decompensated chronic liver disease, liver transplant recipients, sepsis, or hematological disorders. They observed that patients with hematological disorders had a significantly longer filter lifespan (mean = 21.7 h ± 19.7 h), whereas the filter lifespan in all other groups was shorter, with an average duration of less than 12 h. Apart from the hematological group, no other factors, including patient demographics (age, sex, and weight), Apache II score, anticoagulation, type of fluid used (lactate or bicarbonate), or the rate of ultrafiltration and fluid replacement, had a significant impact on the duration of the CRRT circuit. Meanwhile, Chua et al. (86) found that patients with elevated baseline APTT or serum bilirubin, those not mechanically ventilated, or those experiencing peri-circuit thrombocytopenia or a higher international normalized ratio had prolonged filter lifespan. Nonetheless, these patients also experienced more bleeding complications. Furthermore, research (87) indicates that anemia and the need for blood transfusions are prevalent among critically ill patients requiring CRRT for AKI, with an incidence rate reaching 50%. In the study, it was observed that a shorter (<20 h) vs. a longer CRRT filter lifespan was not associated with an increased requirement for packed red blood cell (PRBC) transfusions. However, Sun et al. (88) prospective study results clearly demonstrate that FFP transfusions, both under PRCTP and DTP, have a significant impact on reducing APTT(96.62 ± 42.10 vs. 55.30 ± 29.91, *p* < 0.001, 106.30 ± 63.90 vs. 56.97 ± 42.08, *p* = 0.001), indicating an increase in CRRT circuit clotting risk. The effect of platelet transfusions on APTT is significant under PRCTP but not under DTP, potentially due to the small sample size (74.76 ± 50.49 vs. 71.06 ± 52.24, *p* = 0.016; 49.70 (45.33, 147.68) vs. 51.10 (39.30, 148.33), *p* = 0.564). Overall, FFP appears to have a more consistent and pronounced effect on APTT reduction compared to platelet transfusions, highlighting its importance in managing anticoagulation strategies in patients requiring blood product transfusions. Therefore, during CRRT treatment, it is imperative to choose an appropriate anticoagulation strategy based on the patient’s clinical characteristics to minimize the occurrence of adverse reactions and administer blood transfusion promptly as needed.

CRRT is a commonly utilized technique in the ICU. However, patients undergoing CRRT often face issues of restricted mobility, particularly those utilizing femoral vein catheters. Over the past decade, research has consistently demonstrated that early mobilization, encompassing passive positioning, low-level activities, such as sitting on the edge of the bed and shifting positions, and high-level activities, such as standing and in-place movements, is safe and viable for ICU patients undergoing CRRT (89–92). However, does mobilization during CRRT impact filter pressure, what is its safety profile, and does it affect the lifespan of the filter? In a prospective cohort study led by Wang, involving 33 patients, no instances of filter occlusion or failure were observed during any of the mobilizations, and there were no reported adverse events. The mobilization group exhibited a significantly prolonged filter lifespan compared to the control group (regression coefficient = 13.8, robust 95% confidence interval (CI) = 5.0 to 22.6, *p* = 0.003). Sensitivity analyses indicated that patients undergoing more position changes experienced extended filter life (regression coefficient = 2.0, robust 95% CI = 0.6 to 3.5, *p* = 0.007) (93). Early mobilization, particularly activities within and around the hospital bed, seems to be safe and largely feasible for ICU patients undergoing CRRT (94, 95). Currently, there is a lack of substantial large-scale multicenter studies to validate the research findings and further reinforce conclusions, especially those related to filter lifespan.

## 5 Conclusion

CRRT is crucial in ICU treatment, where circuit lifespan profoundly impacts patient outcomes. This review synthesizes evidence on factors affecting CRRT longevity, focusing on pharmacological and non-pharmacological strategies. RCA reduces bleeding risks while maintaining filter lifespan. UFH is cost-effective, particularly in COVID-19, with rapid reversibility benefits. LMWH may benefit COVID-19 patients with prolonged treatment. NM is effective for high-bleeding risk patients. Catheter selection and placement in femoral or right jugular veins are critical for circuit patency. Surface-modified dual-lumen catheters offer advantages. Material composition, such as polysulfone membranes, influences filter lifespan. CVVHD or CVVHDF modalities extend filter life. Strategic management of effluent volume, blood flow, and downtime is crucial. Simulation-based education and specialized teams improve outcomes. Disease condition impacts filter lifespan; COVID-19 reduces it, while hematological disorders extend it. Early mobilization during CRRT is safe and feasible, potentially extending filter life. Larger studies are needed. This review guides clinical practices to improve outcomes and reduce costs. Future research should innovate CRRT approaches to enhance efficacy and safety.
